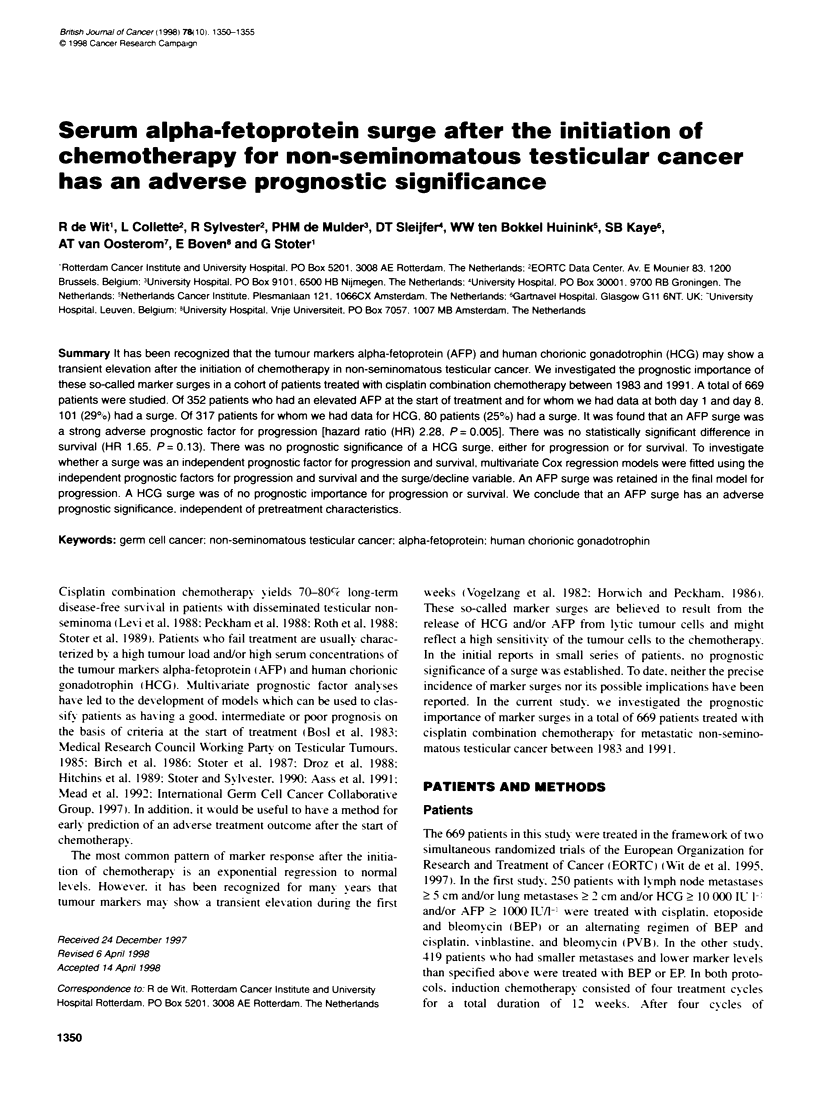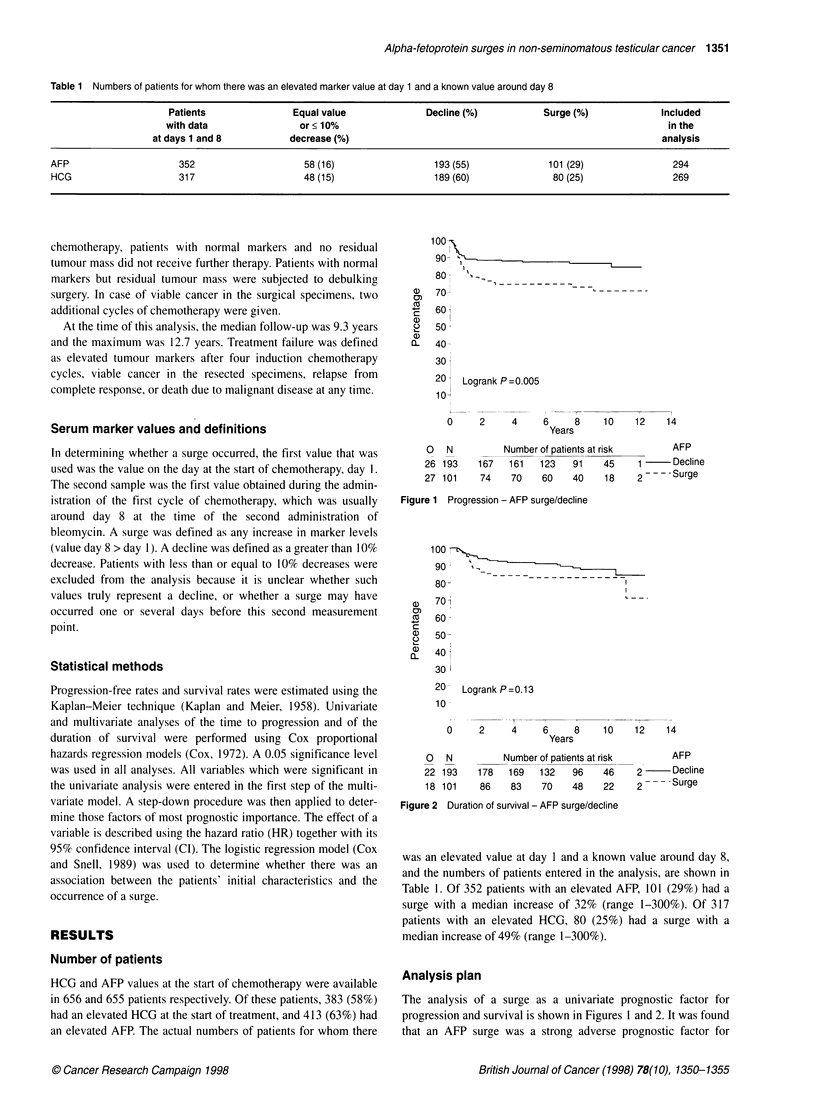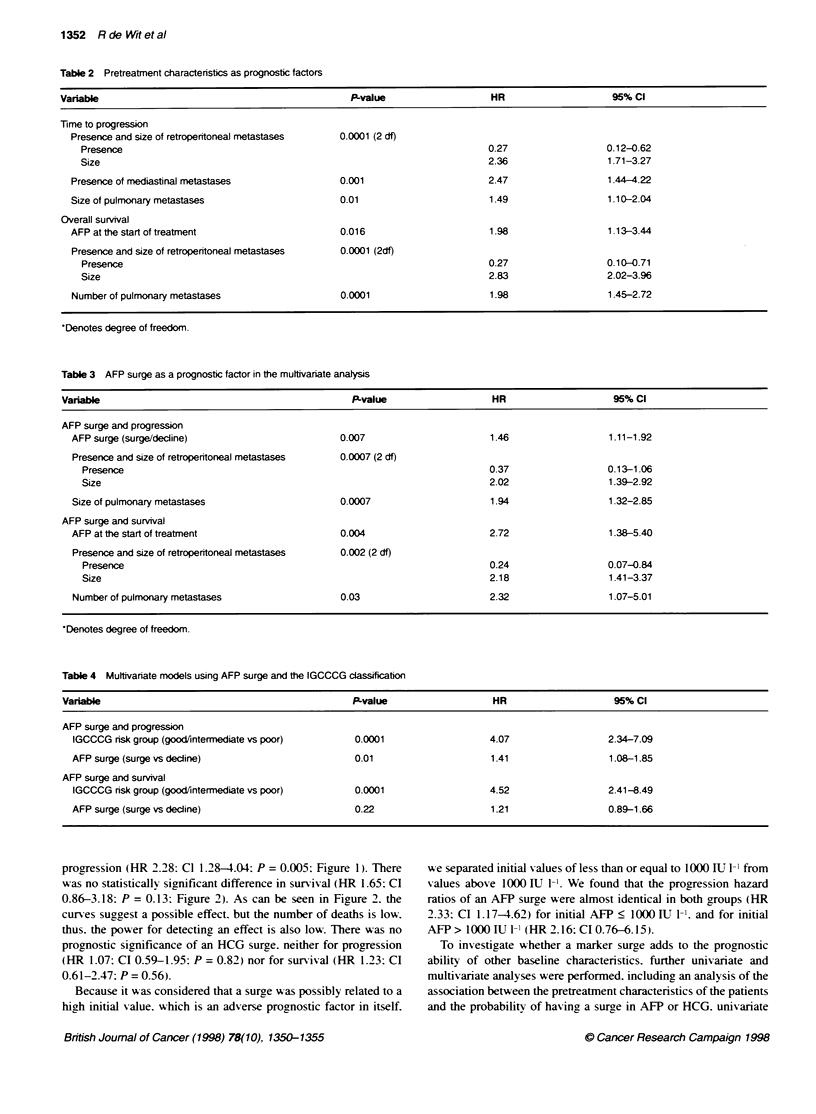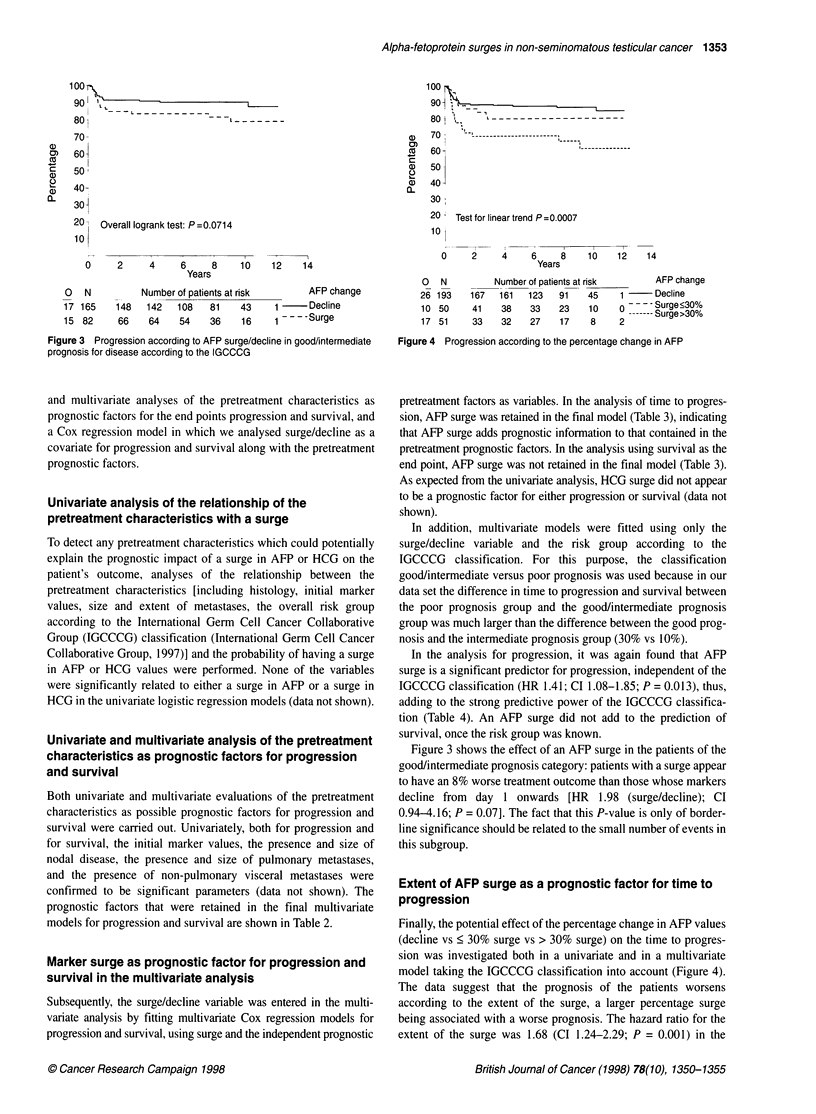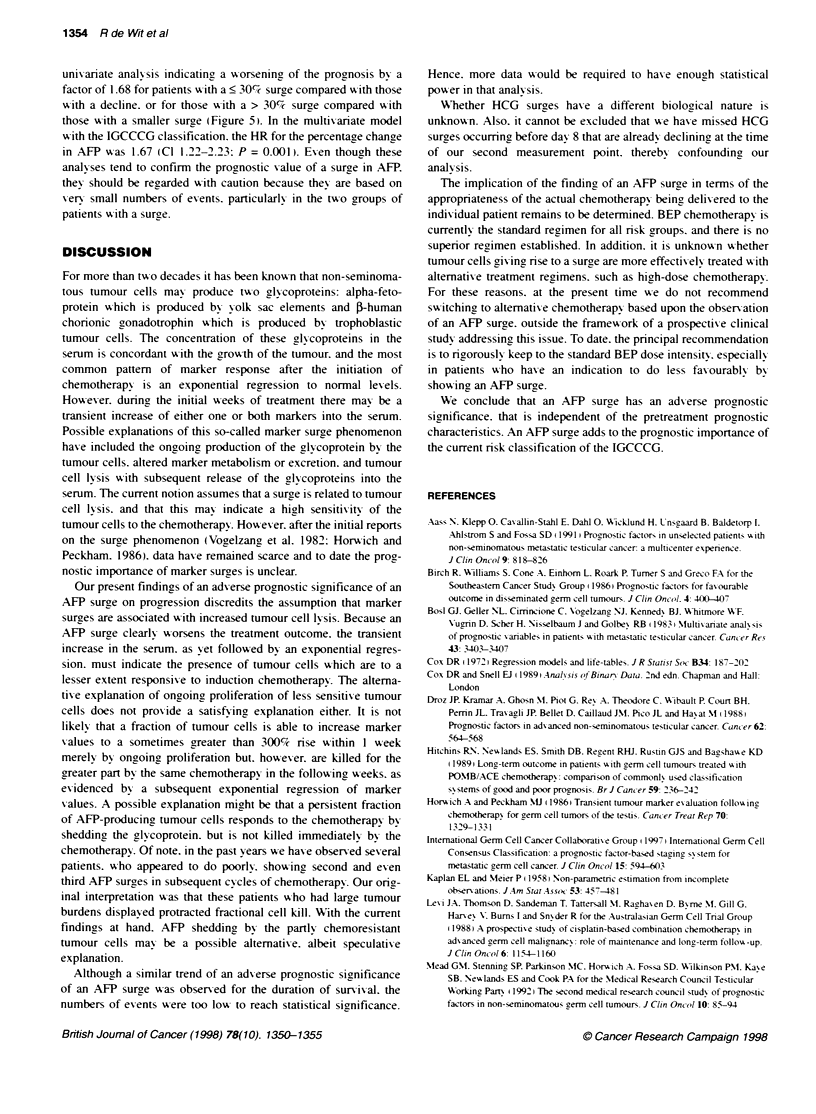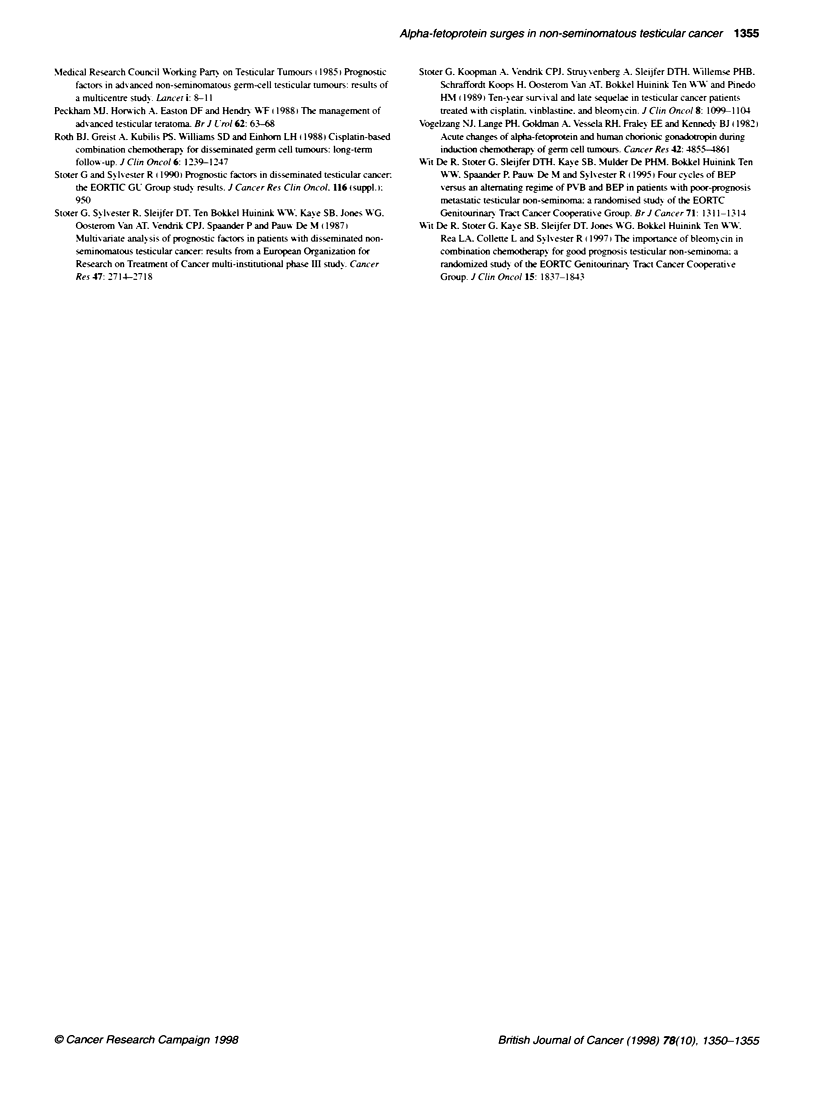# Serum alpha-fetoprotein surge after the initiation of chemotherapy for non-seminomatous testicular cancer has an adverse prognostic significance.

**DOI:** 10.1038/bjc.1998.683

**Published:** 1998-11

**Authors:** R. de Wit, L. Collette, R. Sylvester, P. H. de Mulder, D. T. Sleijfer, W. W. ten Bokkel Huinink, S. B. Kaye, A. T. van Oosterom, E. Boven, G. Stoter

**Affiliations:** Rotterdam Cancer Institute and University Hospital, The Netherlands.

## Abstract

It has been recognized that the tumour markers alpha-fetoprotein (AFP) and human chorionic gonadotrophin (HCG) may show a transient elevation after the initiation of chemotherapy in non-seminomatous testicular cancer. We investigated the prognostic importance of these so-called marker surges in a cohort of patients treated with cisplatin combination chemotherapy between 1983 and 1991. A total of 669 patients were studied. Of 352 patients who had an elevated AFP at the start of treatment and for whom we had data at both day 1 and day 8, 101 (29%) had a surge. Of 317 patients for whom we had data for HCG, 80 patients (25%) had a surge. It was found that an AFP surge was a strong adverse prognostic factor for progression [hazard ratio (HR) 2.28, P=0.005]. There was no statistically significant difference in survival (HR 1.65, P=0.13). There was no prognostic significance of a HCG surge, either for progression or for survival. To investigate whether a surge was an independent prognostic factor for progression and survival, multivariate Cox regression models were fitted using the independent prognostic factors for progression and survival and the surge/decline variable. An AFP surge was retained in the final model for progression. A HCG surge was of no prognostic importance for progression or survival. We conclude that an AFP surge has an adverse prognostic significance, independent of pretreatment characteristics.


British Journal of Cancer (1998) 78(10). 1350–1355
© 1998 Cancer Research Campaign

### Serum alpha-fetoprotein surge after the initiation of chemotherapy for non-seminomatous testicular cancer has an adverse prognostic significance

**R de Wit[1], L Collette[2], R Sylvester[2], PHM de Mulder[3], DT Sleijfer[4], WW ten Bokkel Huinink[5], SB Kaye[6], AT van Oosterom[7], E Boven[8] and G Stoter[1]**

[1]Rotterdam Cancer Institute and University Hospital. PO Box 5201. 3008 AE Rotterdam. The Netherlands: [2]EORTC Data Center. Av. E Mounier 83. 1200 Brussels. Belgium: [3]University Hospital. PO Box 9101. 6500 HB Nijmegen. The Netherlands: [4]University Hospital. PO Box 30001. 9700 RB Groningen. The Netherlands: [5]Netherlands Cancer Institute. Plesmanlaan 121. 1066CX Amsterdam. The Netherlands: [6]Gartnavel Hospital. Glasgow G11 6NT. UK: [7]University Hospital. Leuven. Belgium: [8]University Hospital. Vrije Universiteit. PO Box 7057. 1007 MB Amsterdam. The Netherlands

**Summary** It has been recognized that the tumour markers alpha-fetoprotein (AFP) and human chorionic gonadotrophin (HCG) may show a transient elevation after the initiation of chemotherapy in non-seminomatous testicular cancer. We investigated the prognostic importance of these so-called marker surges in a cohort of patients treated with cisplatin combination chemotherapy between 1983 and 1991. A total of 669 patients were studied. Of 352 patients who had an elevated AFP at the start of treatment and for whom we had data at both day 1 and day 8. 101 (29%) had a surge. Of 317 patients for whom we had data for HCG. 80 patients (25%) had a surge. It was found that an AFP surge was a strong adverse prognostic factor for progression [hazard ratio (HR) 2.28, $P = 0.005$]. There was no statistically significant difference in survival (HR 1.65, $P = 0.13$). There was no prognostic significance of a HCG surge, either for progression or for survival. To investigate whether a surge was an independent prognostic factor for progression and survival, multivariate Cox regression models were fitted using the independent prognostic factors for progression and survival and the surge/decline variable. An AFP surge was retained in the final model for progression. A HCG surge was of no prognostic importance for progression or survival. We conclude that an AFP surge has an adverse prognostic significance, independent of pretreatment characteristics.

**Keywords:** germ cell cancer: non-seminomatous testicular cancer: alpha-fetoprotein: human chorionic gonadotrophin

Cisplatin combination chemotherapy yields 70–80% long-term disease-free survival in patients with disseminated testicular non-seminoma (Levi et al. 1988: Peckham et al. 1988: Roth et al. 1988: Stoter et al. 1989). Patients who fail treatment are usually characterized by a high tumour load and/or high serum concentrations of the tumour markers alpha-fetoprotein (AFP) and human chorionic gonadotrophin (HCG). Multivariate prognostic factor analyses have led to the development of models which can be used to classify patients as having a good, intermediate or poor prognosis on the basis of criteria at the start of treatment (Bosl et al. 1983: Medical Research Council Working Party on Testicular Tumours. 1985: Birch et al. 1986: Stoter et al. 1987: Droz et al. 1988: Hitchins et al. 1989: Stoter and Sylvester. 1990: Aass et al. 1991: Mead et al. 1992: International Germ Cell Cancer Collaborative Group. 1997). In addition, it would be useful to have a method for early prediction of an adverse treatment outcome after the start of chemotherapy.

The most common pattern of marker response after the initiation of chemotherapy is an exponential regression to normal levels. However, it has been recognized for many years that tumour markers may show a transient elevation during the first weeks (Vogelzang et al. 1982: Horwich and Peckham. 1986). These so-called marker surges are believed to result from the release of HCG and/or AFP from lytic tumour cells and might reflect a high sensitivity of the tumour cells to the chemotherapy. In the initial reports in small series of patients, no prognostic significance of a surge was established. To date, neither the precise incidence of marker surges nor its possible implications have been reported. In the current study, we investigated the prognostic importance of marker surges in a total of 669 patients treated with cisplatin combination chemotherapy for metastatic non-seminomatous testicular cancer between 1983 and 1991.

*Received 24 December 1997*
*Revised 6 April 1998*
*Accepted 14 April 1998*

*Correspondence to:* R de Wit. Rotterdam Cancer Institute and University Hospital Rotterdam. PO Box 5201. 3008 AE Rotterdam. The Netherlands

### PATIENTS AND METHODS

### Patients

The 669 patients in this study were treated in the framework of two simultaneous randomized trials of the European Organization for Research and Treatment of Cancer (EORTC) (Wit de et al. 1995. 1997). In the first study, 250 patients with lymph node metastases ≥ 5 cm and/or lung metastases ≥ 2 cm and/or HCG ≥ 10 000 IU $l^{-1}$ and/or AFP ≥ 1000 IU/$l^{-1}$ were treated with cisplatin, etoposide and bleomycin (BEP) or an alternating regimen of BEP and cisplatin, vinblastine, and bleomycin (PVB). In the other study, 419 patients who had smaller metastases and lower marker levels than specified above were treated with BEP or EP. In both protocols, induction chemotherapy consisted of four treatment cycles for a total duration of 12 weeks. After four cycles of

**Table 1** Numbers of patients for whom there was an elevated marker value at day 1 and a known value around day 8

| | Patients with data at days 1 and 8 | Equal value or ≤ 10% decrease (%) | Decline (%) | Surge (%) | Included in the analysis |
|---|---|---|---|---|---|
| AFP | 352 | 58 (16) | 193 (55) | 101 (29) | 294 |
| HCG | 317 | 48 (15) | 189 (60) | 80 (25) | 269 |

chemotherapy, patients with normal markers and no residual tumour mass did not receive further therapy. Patients with normal markers but residual tumour mass were subjected to debulking surgery. In case of viable cancer in the surgical specimens, two additional cycles of chemotherapy were given.

At the time of this analysis, the median follow-up was 9.3 years and the maximum was 12.7 years. Treatment failure was defined as elevated tumour markers after four induction chemotherapy cycles, viable cancer in the resected specimens, relapse from complete response, or death due to malignant disease at any time.

### Serum marker values and definitions

In determining whether a surge occurred, the first value that was used was the value on the day at the start of chemotherapy, day 1. The second sample was the first value obtained during the administration of the first cycle of chemotherapy, which was usually around day 8 at the time of the second administration of bleomycin. A surge was defined as any increase in marker levels (value day 8 > day 1). A decline was defined as a greater than 10% decrease. Patients with less than or equal to 10% decreases were excluded from the analysis because it is unclear whether such values truly represent a decline, or whether a surge may have occurred one or several days before this second measurement point.

### Statistical methods

Progression-free rates and survival rates were estimated using the Kaplan–Meier technique (Kaplan and Meier, 1958). Univariate and multivariate analyses of the time to progression and of the duration of survival were performed using Cox proportional hazards regression models (Cox, 1972). A 0.05 significance level was used in all analyses. All variables which were significant in the univariate analysis were entered in the first step of the multivariate model. A step-down procedure was then applied to determine those factors of most prognostic importance. The effect of a variable is described using the hazard ratio (HR) together with its 95% confidence interval (CI). The logistic regression model (Cox and Snell, 1989) was used to determine whether there was an association between the patients' initial characteristics and the occurrence of a surge.

### RESULTS

### Number of patients

HCG and AFP values at the start of chemotherapy were available in 656 and 655 patients respectively. Of these patients, 383 (58%) had an elevated HCG at the start of treatment, and 413 (63%) had an elevated AFP. The actual numbers of patients for whom there was an elevated value at day 1 and a known value around day 8, and the numbers of patients entered in the analysis, are shown in Table 1. Of 352 patients with an elevated AFP, 101 (29%) had a surge with a median increase of 32% (range 1–300%). Of 317 patients with an elevated HCG, 80 (25%) had a surge with a median increase of 49% (range 1–300%).

| O | N | Number of patients at risk | | | | | AFP |
|---|---|---|---|---|---|---|---|
| 26 | 193 | 167 | 161 | 123 | 91 | 45 | 1 —— Decline |
| 27 | 101 | 74 | 70 | 60 | 40 | 18 | 2 - - - Surge |

**Figure 1** Progression – AFP surge/decline

| O | N | Number of patients at risk | | | | | AFP |
|---|---|---|---|---|---|---|---|
| 22 | 193 | 178 | 169 | 132 | 96 | 46 | 2 —— Decline |
| 18 | 101 | 86 | 83 | 70 | 48 | 22 | 2 - - - Surge |

**Figure 2** Duration of survival – AFP surge/decline

### Analysis plan

The analysis of a surge as a univariate prognostic factor for progression and survival is shown in Figures 1 and 2. It was found that an AFP surge was a strong adverse prognostic factor for

© Cancer Research Campaign 1998

**Table 2** Pretreatment characteristics as prognostic factors

| Variable | P-value | HR | 95% CI |
|---|---|---|---|
| Time to progression | | | |
| Presence and size of retroperitoneal metastases | 0.0001 (2 df) | | |
| Presence | | 0.27 | 0.12–0.62 |
| Size | | 2.36 | 1.71–3.27 |
| Presence of mediastinal metastases | 0.001 | 2.47 | 1.44–4.22 |
| Size of pulmonary metastases | 0.01 | 1.49 | 1.10–2.04 |
| Overall survival | | | |
| AFP at the start of treatment | 0.016 | 1.98 | 1.13–3.44 |
| Presence and size of retroperitoneal metastases | 0.0001 (2df) | | |
| Presence | | 0.27 | 0.10–0.71 |
| Size | | 2.83 | 2.02–3.96 |
| Number of pulmonary metastases | 0.0001 | 1.98 | 1.45–2.72 |

*Denotes degree of freedom.

**Table 3** AFP surge as a prognostic factor in the multivariate analysis

| Variable | P-value | HR | 95% CI |
|---|---|---|---|
| AFP surge and progression | | | |
| AFP surge (surge/decline) | 0.007 | 1.46 | 1.11–1.92 |
| Presence and size of retroperitoneal metastases | 0.0007 (2 df) | | |
| Presence | | 0.37 | 0.13–1.06 |
| Size | | 2.02 | 1.39–2.92 |
| Size of pulmonary metastases | 0.0007 | 1.94 | 1.32–2.85 |
| AFP surge and survival | | | |
| AFP at the start of treatment | 0.004 | 2.72 | 1.38–5.40 |
| Presence and size of retroperitoneal metastases | 0.002 (2 df) | | |
| Presence | | 0.24 | 0.07–0.84 |
| Size | | 2.18 | 1.41–3.37 |
| Number of pulmonary metastases | 0.03 | 2.32 | 1.07–5.01 |

*Denotes degree of freedom.

**Table 4** Multivariate models using AFP surge and the IGCCCG classification

| Variable | P-value | HR | 95% CI |
|---|---|---|---|
| AFP surge and progression | | | |
| IGCCCG risk group (good/intermediate vs poor) | 0.0001 | 4.07 | 2.34–7.09 |
| AFP surge (surge vs decline) | 0.01 | 1.41 | 1.08–1.85 |
| AFP surge and survival | | | |
| IGCCCG risk group (good/intermediate vs poor) | 0.0001 | 4.52 | 2.41–8.49 |
| AFP surge (surge vs decline) | 0.22 | 1.21 | 0.89–1.66 |

progression (HR 2.28; Cl 1.28–4.04; $P = 0.005$; Figure 1). There was no statistically significant difference in survival (HR 1.65; CI 0.86–3.18; $P = 0.13$; Figure 2). As can be seen in Figure 2, the curves suggest a possible effect, but the number of deaths is low, thus, the power for detecting an effect is also low. There was no prognostic significance of an HCG surge, neither for progression (HR 1.07; CI 0.59–1.95; $P = 0.82$) nor for survival (HR 1.23; CI 0.61–2.47; $P = 0.56$).

Because it was considered that a surge was possibly related to a high initial value, which is an adverse prognostic factor in itself, we separated initial values of less than or equal to 1000 IU $l^{-1}$ from values above 1000 IU $l^{-1}$. We found that the progression hazard ratios of an AFP surge were almost identical in both groups (HR 2.33; CI 1.17–4.62) for initial AFP $\leq$ 1000 IU $l^{-1}$, and for initial AFP > 1000 IU $l^{-1}$ (HR 2.16; CI 0.76–6.15).

To investigate whether a marker surge adds to the prognostic ability of other baseline characteristics, further univariate and multivariate analyses were performed, including an analysis of the association between the pretreatment characteristics of the patients and the probability of having a surge in AFP or HCG, univariate

*© Cancer Research Campaign 1998*

**Figure 3** Progression according to AFP surge/decline in good/intermediate prognosis for disease according to the IGCCCG

**Figure 4** Progression according to the percentage change in AFP

and multivariate analyses of the pretreatment characteristics as prognostic factors for the end points progression and survival, and a Cox regression model in which we analysed surge/decline as a covariate for progression and survival along with the pretreatment prognostic factors.

### Univariate analysis of the relationship of the pretreatment characteristics with a surge

To detect any pretreatment characteristics which could potentially explain the prognostic impact of a surge in AFP or HCG on the patient's outcome, analyses of the relationship between the pretreatment characteristics [including histology, initial marker values, size and extent of metastases, the overall risk group according to the International Germ Cell Cancer Collaborative Group (IGCCCG) classification (International Germ Cell Cancer Collaborative Group, 1997)] and the probability of having a surge in AFP or HCG values were performed. None of the variables were significantly related to either a surge in AFP or a surge in HCG in the univariate logistic regression models (data not shown).

### Univariate and multivariate analysis of the pretreatment characteristics as prognostic factors for progression and survival

Both univariate and multivariate evaluations of the pretreatment characteristics as possible prognostic factors for progression and survival were carried out. Univariately, both for progression and for survival, the initial marker values, the presence and size of nodal disease, the presence and size of pulmonary metastases, and the presence of non-pulmonary visceral metastases were confirmed to be significant parameters (data not shown). The prognostic factors that were retained in the final multivariate models for progression and survival are shown in Table 2.

### Marker surge as prognostic factor for progression and survival in the multivariate analysis

Subsequently, the surge/decline variable was entered in the multivariate analysis by fitting multivariate Cox regression models for progression and survival, using surge and the independent prognostic pretreatment factors as variables. In the analysis of time to progression, AFP surge was retained in the final model (Table 3), indicating that AFP surge adds prognostic information to that contained in the pretreatment prognostic factors. In the analysis using survival as the end point, AFP surge was not retained in the final model (Table 3). As expected from the univariate analysis, HCG surge did not appear to be a prognostic factor for either progression or survival (data not shown).

In addition, multivariate models were fitted using only the surge/decline variable and the risk group according to the IGCCCG classification. For this purpose, the classification good/intermediate versus poor prognosis was used because in our data set the difference in time to progression and survival between the poor prognosis group and the good/intermediate prognosis group was much larger than the difference between the good prognosis and the intermediate prognosis group (30% vs 10%).

In the analysis for progression, it was again found that AFP surge is a significant predictor for progression, independent of the IGCCCG classification (HR 1.41; CI 1.08–1.85; $P = 0.013$), thus, adding to the strong predictive power of the IGCCCG classification (Table 4). An AFP surge did not add to the prediction of survival, once the risk group was known.

Figure 3 shows the effect of an AFP surge in the patients of the good/intermediate prognosis category: patients with a surge appear to have an 8% worse treatment outcome than those whose markers decline from day 1 onwards [HR 1.98 (surge/decline); CI 0.94–4.16; $P = 0.07$]. The fact that this $P$-value is only of borderline significance should be related to the small number of events in this subgroup.

### Extent of AFP surge as a prognostic factor for time to progression

Finally, the potential effect of the percentage change in AFP values (decline vs ≤ 30% surge vs > 30% surge) on the time to progression was investigated both in a univariate and in a multivariate model taking the IGCCCG classification into account (Figure 4). The data suggest that the prognosis of the patients worsens according to the extent of the surge, a larger percentage surge being associated with a worse prognosis. The hazard ratio for the extent of the surge was 1.68 (CI 1.24–2.29; $P = 0.001$) in the

© Cancer Research Campaign 1998 

univariate analysis indicating a worsening of the prognosis by a factor of 1.68 for patients with a ≤ 30% surge compared with those with a decline, or for those with a > 30% surge compared with those with a smaller surge (Figure 5). In the multivariate model with the IGCCCG classification, the HR for the percentage change in AFP was 1.67 (Cl 1.22–2.23; $P = 0.001$). Even though these analyses tend to confirm the prognostic value of a surge in AFP, they should be regarded with caution because they are based on very small numbers of events, particularly in the two groups of patients with a surge.

### DISCUSSION

For more than two decades it has been known that non-seminomatous tumour cells may produce two glycoproteins: alpha-fetoprotein which is produced by yolk sac elements and β-human chorionic gonadotrophin which is produced by trophoblastic tumour cells. The concentration of these glycoproteins in the serum is concordant with the growth of the tumour, and the most common pattern of marker response after the initiation of chemotherapy is an exponential regression to normal levels. However, during the initial weeks of treatment there may be a transient increase of either one or both markers into the serum. Possible explanations of this so-called marker surge phenomenon have included the ongoing production of the glycoprotein by the tumour cells, altered marker metabolism or excretion, and tumour cell lysis with subsequent release of the glycoproteins into the serum. The current notion assumes that a surge is related to tumour cell lysis, and that this may indicate a high sensitivity of the tumour cells to the chemotherapy. However, after the initial reports on the surge phenomenon (Vogelzang et al, 1982; Horwich and Peckham, 1986), data have remained scarce and to date the prognostic importance of marker surges is unclear.

Our present findings of an adverse prognostic significance of an AFP surge on progression discredits the assumption that marker surges are associated with increased tumour cell lysis. Because an AFP surge clearly worsens the treatment outcome, the transient increase in the serum, as yet followed by an exponential regression, must indicate the presence of tumour cells which are to a lesser extent responsive to induction chemotherapy. The alternative explanation of ongoing proliferation of less sensitive tumour cells does not provide a satisfying explanation either. It is not likely that a fraction of tumour cells is able to increase marker values to a sometimes greater than 300% rise within 1 week merely by ongoing proliferation but, however, are killed for the greater part by the same chemotherapy in the following weeks, as evidenced by a subsequent exponential regression of marker values. A possible explanation might be that a persistent fraction of AFP-producing tumour cells responds to the chemotherapy by shedding the glycoprotein, but is not killed immediately by the chemotherapy. Of note, in the past years we have observed several patients, who appeared to do poorly, showing second and even third AFP surges in subsequent cycles of chemotherapy. Our original interpretation was that these patients who had large tumour burdens displayed protracted fractional cell kill. With the current findings at hand, AFP shedding by the partly chemoresistant tumour cells may be a possible alternative, albeit speculative explanation.

Although a similar trend of an adverse prognostic significance of an AFP surge was observed for the duration of survival, the numbers of events were too low to reach statistical significance. Hence, more data would be required to have enough statistical power in that analysis.

Whether HCG surges have a different biological nature is unknown. Also, it cannot be excluded that we have missed HCG surges occurring before day 8 that are already declining at the time of our second measurement point, thereby confounding our analysis.

The implication of the finding of an AFP surge in terms of the appropriateness of the actual chemotherapy being delivered to the individual patient remains to be determined. BEP chemotherapy is currently the standard regimen for all risk groups, and there is no superior regimen established. In addition, it is unknown whether tumour cells giving rise to a surge are more effectively treated with alternative treatment regimens, such as high-dose chemotherapy. For these reasons, at the present time we do not recommend switching to alternative chemotherapy based upon the observation of an AFP surge, outside the framework of a prospective clinical study addressing this issue. To date, the principal recommendation is to rigorously keep to the standard BEP dose intensity, especially in patients who have an indication to do less favourably by showing an AFP surge.

We conclude that an AFP surge has an adverse prognostic significance, that is independent of the pretreatment prognostic characteristics. An AFP surge adds to the prognostic importance of the current risk classification of the IGCCCG.

### REFERENCES

Aass N, Klepp O, Cavallin-Stahl E, Dahl O, Wicklund H, Unsgaard B, Baldetorp I, Ahlstrom S and Fossa SD (1991) Prognostic factors in unselected patients with non-seminomatous metastatic testicular cancer: a multicenter experience. *J Clin Oncol* **9**: 818–826

Birch R, Williams S, Cone A, Einhorn L, Roark P, Turner S and Greco FA for the Southeastern Cancer Study Group (1986) Prognostic factors for favourable outcome in disseminated germ cell tumours. *J Clin Oncol*, **4**: 400–407

Bosl GJ, Geller NL, Cirrincione C, Vogelzang NJ, Kennedy BJ, Whitmore WF, Vugrin D, Scher H, Nisselbaum J and Golbey RB (1983) Multivariate analysis of prognostic variables in patients with metastatic testicular cancer. *Cancer Res* **43**: 3403–3407

Cox DR (1972) Regression models and life-tables. *J R Statist Soc* **B34**: 187–202

Cox DR and Snell EJ (1989) *Analysis of Binary Data*. 2nd edn. Chapman and Hall: London

Droz JP, Kramar A, Ghosn M, Piot G, Rey A, Theodore C, Wibault P, Court BH, Perrin JL, Travagli JP, Bellet D, Caillaud JM, Pico JL and Hayat M (1988) Prognostic factors in advanced non-seminomatous testicular cancer. *Cancer* **62**: 564–568

Hitchins RN, Newlands ES, Smith DB, Regent RHJ, Rustin GJS and Bagshawe KD (1989) Long-term outcome in patients with germ cell tumours treated with POMB/ACE chemotherapy: comparison of commonly used classification systems of good and poor prognosis. *Br J Cancer* **59**: 236–242

Horwich A and Peckham MJ (1986) Transient tumour marker evaluation following chemotherapy for germ cell tumors of the testis. *Cancer Treat Rep* **70**: 1329–1331

International Germ Cell Cancer Collaborative Group (1997) International Germ Cell Consensus Classification: a prognostic factor-based staging system for metastatic germ cell cancer. *J Clin Oncol* **15**: 594–603

Kaplan EL and Meier P (1958) Non-parametric estimation from incomplete observations. *J Am Stat Assoc* **53**: 457–481

Levi JA, Thomson D, Sandeman T, Tattersall M, Raghaven D, Byrne M, Gill G, Harvey V, Burns I and Snyder R for the Australasian Germ Cell Trial Group (1988) A prospective study of cisplatin-based combination chemotherapy in advanced germ cell malignancy: role of maintenance and long-term follow-up. *J Clin Oncol* **6**: 1154–1160

Mead GM, Stenning SP, Parkinson MC, Horwich A, Fossa SD, Wilkinson PM, Kaye SB, Newlands ES and Cook PA for the Medical Research Council Testicular Working Party (1992) The second medical research council study of prognostic factors in non-seminomatous germ cell tumours. *J Clin Oncol* **10**: 85–94

 © Cancer Research Campaign 1998

Medical Research Council Working Party on Testicular Tumours (1985) Prognostic factors in advanced non-seminomatous germ-cell testicular tumours: results of a multicentre study. *Lancet* **i**: 8–11

Peckham MJ. Horwich A. Easton DF and Hendry WF (1988) The management of advanced testicular teratoma. *Br J Urol* **62**: 63–68

Roth BJ. Greist A. Kubilis PS. Williams SD and Einhorn LH (1988) Cisplatin-based combination chemotherapy for disseminated germ cell tumours: long-term follow-up. *J Clin Oncol* **6**: 1239–1247

Stoter G and Sylvester R (1990) Prognostic factors in disseminated testicular cancer: the EORTIC GU Group study results. *J Cancer Res Clin Oncol.* **116** (suppl.): 950

Stoter G. Sylvester R. Sleijfer DT. Ten Bokkel Huinink WW. Kaye SB. Jones WG. Oosterom Van AT. Vendrik CPJ. Spaander P and Pauw De M (1987) Multivariate analysis of prognostic factors in patients with disseminated non-seminomatous testicular cancer: results from a European Organization for Research on Treatment of Cancer multi-institutional phase III study. *Cancer Res* **47**: 2714–2718

Stoter G. Koopman A. Vendrik CPJ. Struyvenberg A. Sleijfer DTH. Willemse PHB. Schraffordt Koops H. Oosterom Van AT. Bokkel Huinink Ten WW and Pinedo HM (1989) Ten-year survival and late sequelae in testicular cancer patients treated with cisplatin. vinblastine. and bleomycin. *J Clin Oncol* **8**: 1099–1104

Vogelzang NJ. Lange PH. Goldman A. Vessela RH. Fraley EE and Kennedy BJ (1982) Acute changes of alpha-fetoprotein and human chorionic gonadotropin during induction chemotherapy of germ cell tumours. *Cancer Res* **42**: 4855–4861

Wit De R. Stoter G. Sleijfer DTH. Kaye SB. Mulder De PHM. Bokkel Huinink Ten WW. Spaander P. Pauw De M and Sylvester R (1995) Four cycles of BEP versus an alternating regime of PVB and BEP in patients with poor-prognosis metastatic testicular non-seminoma: a randomised study of the EORTC Genitourinary Tract Cancer Cooperative Group. *Br J Cancer* **71**: 1311–1314

Wit De R. Stoter G. Kaye SB. Sleijfer DT. Jones WG. Bokkel Huinink Ten WW. Rea LA. Collette L and Sylvester R (1997) The importance of bleomycin in combination chemotherapy for good prognosis testicular non-seminoma: a randomized study of the EORTC Genitourinary Tract Cancer Cooperative Group. *J Clin Oncol* **15**: 1837–1843

© Cancer Research Campaign 1998